# Observational Evidence of the Association Between Handgrip Strength, Hand Dexterity, and Cognitive Performance in Community-Dwelling Older Adults: A Systematic Review

**DOI:** 10.2188/jea.JE20170041

**Published:** 2018-09-05

**Authors:** Kimi Estela Kobayashi-Cuya, Ryota Sakurai, Hiroyuki Suzuki, Susumu Ogawa, Toru Takebayashi, Yoshinori Fujiwara

**Affiliations:** 1 Research Team for Social Participation and Community Health, Tokyo Metropolitan Institute of Gerontology, Tokyo, Japan; 2 Department of Preventive Medicine and Public Health, School of Medicine, Keio University, Tokyo, Japan

**Keywords:** hand motor function, handgrip strength, hand dexterity, cognitive function, community-dwelling older adults

## Abstract

**Background:**

Deterioration of hand motor function is a possible risk factor of cognitive impairment in older adults. Despite a growing body of research, a lack of clarity exists regarding the relationships. This review offers a synthesis of existing observational studies evaluating the associations of handgrip strength and hand dexterity with cognitive performance in community-dwelling older adults.

**Methods:**

PubMed, PsycINFO, and ScienceDirect were systematically searched (search dates: 1990–2016), and relevant articles were cross-checked for related and relevant publications.

**Results:**

Twenty-two observational studies assessed the association of handgrip strength or hand dexterity with cognitive performance; none evaluated handgrip strength and hand dexterity together. Handgrip strength was associated with global cognition, mostly assessed using the Mini-Mental State Examination, cross-sectionally and longitudinally. Also, one cross-sectional and three longitudinal studies found an association with cognitive domains, such as language, memory, visuospatial ability, working memory, and processing speed. Hand dexterity was only assessed cross-sectionally in four studies. These studies found an association with cognitive domains, such as executive function.

**Conclusions:**

Although handgrip strength was associated with cognitive performance, it is unclear which variable at baseline affects the other in the long-term. Cross-sectional studies indicate an association between hand dexterity and cognitive performance, yet longitudinal studies are needed to elucidate this association. The interaction effects of both decreased grip strength and hand dexterity on cognitive performance is still unclear; therefore, future studies will need to consider the interaction of the three variables cross-sectionally and longitudinally.

## INTRODUCTION

Cognitive impairment in late adulthood is one of the causes leading to loss of independence^1^^,^^2^ and dementia,^3^^,^^4^ which in turn represents an economic burden to the national public health and social welfare.^5^ Several studies in public health and epidemiology have aimed to understand the causes leading to this condition in the older population,^6^^,^^7^ its early detection,^8^ and how to prevent it from worsening.^9^ Cognitive decline has been related to the aging process^10^^–^^12^; however, there is still a thin line between what is considered normal and pathological cognitive aging.^13^^,^^14^ Identification of measurable indicators associated with cognitive impairment in healthy older adults would contribute to the early detection—and possibly prevention—of pathological cognitive decline, such as mild cognitive impairment (MCI), and would foster intervention programs aiming to maintain cognitive processes related to independent living in older adults.

Behavioral research^15^^,^^16^ has recently paid much attention to impairment of gait function as a potential motor risk factor for impairment in cognitive function; however, decreased hand motor function, similar to gait function, is also a possible candidate risk factor of cognitive impairment because of its association with cortical brain activity.^17^^–^^22^ This association may rely on common neural processes shared between the cognitive and motor areas of the central nervous system and the motor neurons of the peripheral nervous system^23^^,^^24^; in other words, it can be hypothesized that the neural circuitry of the hand motor function may associate with that of cognitive performance. This is well supported by the findings that Alzheimer’s disease (AD) patients performed hand motor tasks worse than MCI patients who, in turn, performed worse than older adults without cognitive impairment.^10^^,^^25^^,^^26^ However, the association between hand motor function and cognition is still unclear, as the hand is not only limited to activities where strength is needed, but it is also relevant for performing fine and complex activities that require different skills to perform a specific action, such as precision,^27^ speed,^28^ aiming,^29^ and tracing.^30^ Therefore, handgrip strength and hand dexterity, representing hand motor function in this review, seem to be crucial components of functional independence and cognitive maintenance in older adults. This study offers a narrative synthesis of existing observational studies evaluating the association of hand motor function, including handgrip strength and hand dexterity, with cognitive performance in community-dwelling healthy older adults.

## METHODS

### Search strategy

Database searches of PubMed, PsycINFO, and ScienceDirect were conducted using the same search strategy consisting of two combined keyword formulas:

Formula 1 (F1): (elderly OR older adults) AND (handgrip strength OR grip strength OR grasp strength OR grasping power OR grip force) AND (cognitive function)
Formula 2 (F2): (elderly OR older adults) AND (hand dexterity OR dexterity OR hand ability OR manual ability OR motor skill) AND (cognitive function)

A third formula (F3) combining F1 and F2 was considered in a preliminary search conducted in PubMed as the purpose of this review is to examine previous studies evaluating the association between handgrip strength, hand dexterity and cognitive performance. However, only two formulas (F1 and F2) are reported in this review because all findings in F3 were duplicated in F2. The keywords used for handgrip strength were handgrip strength, grip strength, grasp strength, grasping power, and grip force; the keywords used for hand dexterity were hand dexterity, dexterity, hand ability, manual ability, and motor skill. The searches were performed in December 2016, and the selection of the articles were limited to those published in English between January 1990 and December 2016. A grey literature search was performed using Google and Google Scholar. Hand searching through citations and references of relevant articles was also undertaken. The limiters used were year of publication (1990–2016), language (English), population (humans), journal articles (peer reviewed), observational studies (quantitative, qualitative, longitudinal, follow-up, prospective and retrospective), and age (middle aged to ≥80 years old).

### Inclusion and exclusion criteria

#### Population

Studies considering middle-aged subjects and over were included in the review (middle-aged: 45–64 years, aged: ≥65 years, ≥80 years), excluding post-mortem studies. This study reviewed previous observational studies examining the relationship between hand motor function and age-related cognitive impairment, not pathological deficits, among community-dwelling older adults; therefore, studies including patients with neurological disorders were excluded.

#### Study design

This review investigated the observational evidence of the association between age-related changes in fine upper motor performance and cognitive impairment; therefore, intervention studies were not included in the analysis. Observational studies, including cross-sectional studies and longitudinal studies, were included. Case studies—where a cognitive control group was included and whose data was separately analyzed—were also included in this review.

#### Quality assessment

**Figure 1.**
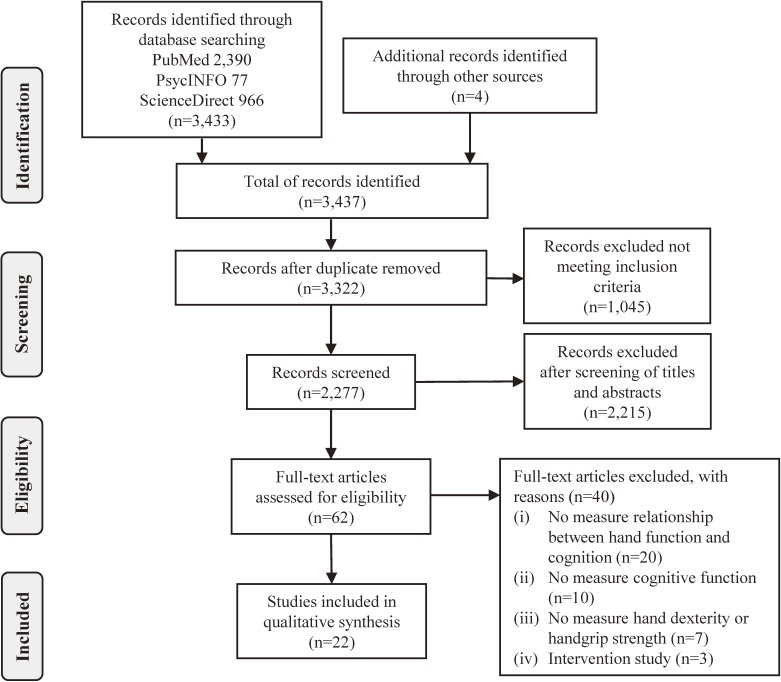
PRISMA flow diagram for the systematic review

Titles and abstracts were independently searched for relevance (KK). The full-text publications of the selected studies were reviewed against the inclusion criteria, and the reasons for exclusion at this stage were recorded (Figure 1). Several meetings with the team members (KK, RS, YF, HS, and SO) were held to discuss, verify, and agree on the selection of the articles.

### Data analysis

An evidence table was constructed to help organize and summarize the information of the studies included in the review. The information extracted was setting, number of participants, anthropometric data, cognitive and hand function measurements. A narrative synthesis was completed due to the different measures of cognition and hand function tests used in the selected studies, which made the body of evidence unsuitable for meta-analysis.

## RESULTS

The search results and reasons for exclusion are presented in the PRISMA flow diagram in Figure 1. After applying the inclusion and exclusion criteria, 62 articles were identified from PubMed, PsycINFO, and ScienceDirect databases. No studies evaluated the interaction between the three variables in question. Instead, 22 articles reported the association in pairs; that is, they reported the associations between handgrip strength and cognitive performance or hand dexterity and cognitive performance (Table 1 and Table 2).

**Table 1.** Cross-sectional studies examining the association between hand and cognitive function

Reference (First author and year of publication)	Dataset, Country	*n* male: M%	Mean (SD) age (range)	Years of education	Cognitive function tests	Hand function tests	Main outcome: Association
Auyeung, 2008^35^	Community-dwelling OA, China	4,000 M: 50%	M: 72.3 (5.0) F: 72.5 (5.3)	(>12 years) M: 13.6% F: 6.0%	CSI-D	Jamar^®^ hydraulic hand dynamometer	The cognitively impaired group had weaker handgrip strength than the non-impaired subjects, in both genders (*P* < 0.001), even after covariate adjustment.
Bramell-Risberg, 2010^31^	GAS, Sweden	1,927 (1,207 non-CI) M: 43.1%	(60–93)	High School (29.6%) University (23.0%)	MMSE and the 3-word delayed recall of the MMSE	Grippit^®^ dynamometer	No significant association between cognitive impairment and averaged handgrip strength in all groups, even after covariate adjustment. No difference in maximum handgrip strength between cases and controls.
Confortin, 2015^36^	Saude-AC, Brazil	270 M: 0%	73.2 (8.8) (60–100)	Not specified	MMSE	Mechanical dynamometer Takei	Prevalence of low handgrip strength significantly correlated with poor performance in the MMSE (*P* < 0.02).
Lin, 2016^33^	Community-dwelling OA, Taiwan	378 (100 SA) M: 53%	75.9 (7.3)	>9 years: (66.7%)	MMSE	Jamar^®^ hydraulic hand dynamometer	The SA group (MMSE ≥24) hand significantly stronger handgrip strength than the non-SA group (MMSE <24) in both genders, even after covariate adjustment (*P* < 0.001).
Malmstrom, 2005^34^	AHA, USA	998 M: 41.8%	56.8 (4.4) (49–65)	12.5 (2.8)	MMSE and Animal Naming test	Baseline^®^ hand dynamometer	Lower MMSE (*P* = 0.001) and animal naming test performance (*P* < 0.05) were associated with weaker handgrip strength, even after covariate adjustment.
Taekema, 2010^37^	Leiden 85-plus Study, The Netherlands	555 M: 35%	85	Not specified	MMSE	Jamar^®^ hydraulic hand dynamometer	Significant association between the lowest handgrip strength tertile and the lowest MMSE scores (*P* < 0.001). Also, lower handgrip strength predicted a decline in cognitive performance (*P* ≤ 0.001).
Takata, 2008^32^	Age-related general and oral health, Japan	205 M: 43.0%	85	9.4 (2.6)	MMSE	Smedley hand dynamometer	Lower MMSE associated with weaker handgrip strength before and after covariate adjustment (*P* < 0.001 vs *P* < 0.01). Subjects with normal cognitive function had stronger handgrip strength.
Ashendorf, 2009^39^	Community-dwelling OA, USA	307 M: 49.0%	M: 63.9 (6.1) F: 64.0 (5.8) (55–74)	M 13.9 (2.7) F 13.6 (2.5)	Memory: LM, VR of WMS; Language: NAART; Executive function: TMT A & B, WCST, SCWT; Visuospatial: BD; Processing speed: DS (WAIS-R)	GPT	GPT performance significantly correlated with all cognitive tasks (*P* < 0.01) in both genders. Women were faster than men in GPT performance.
Bezdicek, 2014^40^	Matched healthy volunteers, Czech Republic	65 (20 non-PD) M: 70.0%	65.5 (8.4) (48–80)	14.3 (2.9)	MoCA	GPT	GPT correlated with MoCA scores (*P* < 0.001). Cases and controls differed in GPT performance (*P* < 0.001) and subtests of MoCA: attention (*P* < 0.01), visuospatial and abstraction (*P* < 0.05).
Rodriguez-Aranda, 2016^28^	Community-dwelling OA, Norway	30 (15 OA) M: 33.0%	74.0 (6.9) (67–93)	13.0 (3.9)	Executive function: TMT, SCWT; Working memory: DSF & DSB (WAIS-R)	PPT	The Stroop task and TMT-B associated with hand movement variability; however, the direction of the association is unclear.
Yozbatiran, 2006^38^	Matched healthy volunteers, Turkey	59 (28 non-MS) M: 28.6%	35.4 (7.2) (23–66)	11.1 (3.1) (11–14)	PASAT	9-HPT	Moderate correlation between 9-HPT and PASAT (*P* < 0.01) in cases and controls. Cognitive function did not predict upper extremity motor function.

OA, older adults; CSI-D, the community screening instrument of dementia; GAS, aging in Skane; CI, cognitive impairment; MMSE, mini-mental state examination; SA, successful aging; AHA, African Americans for humanism; LM, logical memory; VR of WMS, visual reproduction of the Wechsler memory scale; NAART, the North American adult reading test; TMT, trail making test; WCST, Wisconsin card sorting test; SCWT, Stroop color and word Test; BD, block design; DS, digit symbol; WAIS-R, Wechsler adult intelligence scale-revised; GPT, Grooved pegboard test; PD, Parkinson disease; MoCA, Montreal cognitive assessment; DSF, digit span forward; DSB, digit span backward; PPT, Purdue pegboard test; MS, multiple sclerosis; PASAT, paced auditory serial addition test; 9-HPT, nine-hole peg test.

**Table 2.** Longitudinal studies examining the association between hand and cognitive performance

Reference (First author and year of publication)	Dataset, Country	*n* male: M%	Mean (SD) age (range)	Years of education mean (SD)	Cognitive function tests	Hand function tests	Main outcome: Association
Alfaro-Acha, 2006^44^	H-EPESE, USA	2,160 M: 42.5%	71.9 (5.9)	5.3 (3.9)	MMSE	Jamar^®^ hydraulic hand dynamometer	The weakest handgrip strength at baseline significantly correlated with lower MMSE at each follow-up (*P* < 0.001) in both genders and remained significant over 7 years, even after covariate adjustment.
Atkinson, 2010^52^	WHIMS, USA	1,793 M: 0%	70.3 (3.7) (65–80)	<High School (7%)	3MS	Standard hand dynamometer	Baseline 3MS associated with weaker handgrip strength (*P* < 0.05) cross-sectionally and over 6-year follow-up. Baseline handgrip strength did not predict 3MS score changes longitudinally.
Auyeung, 2011^47^	Community-dwelling OA, China	2,737 M: 55%	M: 71.6 (4.6) F: 71.5 (4.9)	M 7.9 (5.1) F 5.0 (5.0)	MMSE	Jamar^®^ hydraulic hand dynamometer	After 4 years of the study, weaker handgrip strength significantly correlated with lower MMSE (*P* < 0.001), after covariate adjustment (*P* < 0.01) in both genders.
Boyle, 2010^46^	The Rush Memory and Aging Project, USA	761 M: 23.6%	79.0 (7.1) (54–100)	14.5 (3.2)	Episodic memory: Story A from LM, EBS, WLM, WLRc, WLRcg; Semantic memory: 15-item BNT, VF, 15-item RT; Visuospatial ability: 15-item JLO, 16-item SPM; Working memory: DSF, DSB, DO; Perceptual speed: SDMT, NC, SNST; MMSE	Jamar^®^ hydraulic hand dynamometer	Baseline weaker handgrip strength associated with the risk of MCI (*P* < 0.01) over 12 years, even after covariate adjustment. Baseline physical frailty associated with a faster decline in global cognition, episodic memory, sematic memory, visuospatial abilities, working memory, and perceptual speed.
Charles, 2006^51^	HHP and HAAS	3,522 100%	52.6 (4.7) (46–68)	10.5 (3.2)	CASI	Smedley hand dynamometer	After 25 years of follow-up, decline in handgrip strength significantly correlated with lower CASI scores (*P* < 0.001).
Deeg, 1992^48^	Koganei Study, Japan	240 M: 45.0%	(69–71)	High School (42%)	The Benton Visual Retention test	Standard hand dynamometer	The visual memory test predicted handgrip strength changes only in men (*P* < 0.05) over 10 years of study.
Praetorius Bjork, 2016^50^	OCTO-Twin Study, Sweden	449 M: 35.6%	83.5 (3.2)	7.3 (2.5)	Episodic memory: MRT, PR, TPM; Semantic memory: IT (modified WAIS); Short-term memory: DSF; Visuospatial ability: BD, FLT; Working memory: DSB; Motor & Perceptual speed: SDSST	Martin vigorimeter	Performance of all cognitive tests: episodic memory, semantic memory, visuospatial ability, perceptual speed (*P* < 0.001); working memory (*P* = 0.001); short-term memory (*P* < 0.01) associated with handgrip strength. The rate of change of episodic memory, semantic memory and visuospatial ability (*P* < 0.01); and short-term memory (*P* = 0.056) associated with grip strength before death.
Raji, 2005^49^	H-EPESE, USA	2,381 M: 43.0%	72.1 (6.0)	4.9 (3.9)	MMSE	Jamar^®^ hydraulic hand dynamometer	Lower MMSE (<21) correlated with weaker handgrip strength (*P* < 0.001) cross-sectionally and over 7-years (*P* < 0.001), even after covariate adjustment.
Samper-Ternent, 2008^45^	H-EPESE, USA	1,370 (684 non-frail) M: 59.0%	73.1 (4.8)	5.9 (4.1)	MMSE	Jamar^®^ hydraulic hand dynamometer	Over 10 years, MMSE scores remained significantly lower in frail subjects than non-frail subjects, even after covariate adjustment (*P* < 0.001). Frail subjects had lower handgrip strength (*P* < 0.001).
Taekema, 2012^53^	Leiden 85-plus Study, The Netherlands	555 35%	85	≤Elementary School (63.6%)	MMSE Memory: 12-PLT; Attention: Abbreviated Stroop test Processing speed: LDST	Jamar^®^ hydraulic hand dynamometer	After 4 years of follow-up, performance of all cognitive tests associated with decline in handgrip strength, even after covariate adjustment (*P* < 0.05). However, baseline handgrip strength only associated with decline in MMSE scores (*P* = 0.007).
Veronese, 2016^57^	Community-dwelling OA, China	1,249 M: 40.5%	72.2 (5.8) (65–96)	>5 years: (19.7%)	MMSE CI: MMSE<24 CD: MMSE 24–27	Jamar^®^ hydraulic hand dynamometer	Unadjusted values showed that baseline weaker handgrip strength associated with cognitive impairment at 4 years of follow-up (*P* < 0.001). However, no association was found after covariate adjustment.

H-EPESE, Hispanic established population for the epidemiological study of the elderly; MMSE, mini-mental state examination; WHIMS, women’s health initiative memory study; 3MS, the modified mini-mental state examination; OA, older adults; LM, logical memory; EBS, the east Boston story; WLM, word list memory; WLRc, word list recall; WLRcg, word list recognition; BNT, the Boston naming test; VF, verbal fluency; RT, reading test; JLO, judgement of line orientation; SPM, standard progressive matrices; DSF, digit span forward; DSB, digit span backward; DO, digit ordering; SDMT, symbol digit modalities test; NC, number comparison; SNST, Stroop neuropsychological screening test; HHP, the Honolulu heart program; HAAS, the Honolulu-Asia aging study; CASI, cognitive abilities screening index instrument; MRT, the memory-in-reality test; PR, prose recall; TPM, Thurstone’s picture memory; IT, information test; WAIS, the Wechsler adult intelligence scale; BD, block design; FLT, the figure logic task; SDSST, speeded digit-symbol substitution test; 12-PLT, 12-picture learning test; LDST, letter digit substitution task; CI, cognitive impairment; CD, cognitive decline.

Of the 22 articles included in the review, 11 were cross-sectional studies (Table 1) and 11 were longitudinal studies (Table 2). Concerning the cross-sectional studies, seven studies included handgrip strength and four studies included hand dexterity, whereas all longitudinal studies included only handgrip strength in their analyses.

Hand dexterity was measured with peg tests (Table 1) and handgrip strength was measured with hand dynamometers, as part of functional health tests and as a composite measure of frailty in some studies (Table 1 and Table 2). MMSE was used as a measurement of global cognitive function and to describe the cohort in some studies.^31^^–^^33^ The cognitive tests used in these studies were categorized in this review by cognitive domains (Table 3) and were commonly assessed using the MMSE.

**Table 3.** Cognitive domains measured in each study (categorized according to each author’s suggestion)

Reference (First author and year of publication)	Study Design	Global	Memory	Language	Executive function	Visuospatial ability (spatial organization)	Attention Concentration Working memory	Processing speed (perceptual speed)
Auyeung, 2008^35^	CS	CSI-D	—	—	—	—	—	—
Bramell-Risberg, 2010^31^	CS	MMSE	3-word delayed recall of the MMSE	—	—	—	—	—
Confortin, 2015^36^	CS	MMSE	—	—	—	—	—	—
Lin, 2016^33^	CS	MMSE	—	—	—	—	—	—
Malmstrom, 2005^34^	CS	MMSE	—	Animal Naming test	—	—	—	—
Taekema, 2010^37^	CS	MMSE	—	—	—	—	—	—
Takata, 2008^32^	CS	MMSE	—	—	—	—	—	—
Ashendorf, 2009^39^	CS	—	Logical Memory & Visual Reproduction (WMS)	NAART	TMT A&B, WCST, SCWT	Block Design (WAIS-R)	Digit Symbol (WAIS-R)	Digit Symbol (WAIS-R)
Bezdicek, 2014^40^	CS	MoCA	—	—	—	—	—	—
Rodriguez-Aranda, 2016^28^	CS	—	—	—	TMT, SCWT	—	DSF & DSB (WAIS-R)	—
Yozbatiran, 2006^38^	CS	—	—	—	—	—	PASAT	PASAT
Alfaro-Acha, 2006^44^	7-year LS	MMSE	—	—	—	—	—	—
Atkinson, 2010^52^	6-year LS	3MS		—	—	—	—	—
Auyeung, 2011^47^	4-year LS	MMSE	—	—	—	—	—	—
Boyle, 2010^46^	12-year LS	MMSE	Story A from LM, EBS, WLM, WLRc, WLRcg, 15-item BNT, VF, 15-item RT	—	—	15-item JLO, 16-item SPM	DSF, DSB, DO	SDMT, NC, SNST
Charles, 2006^51^	25-year LS	CASI	—	—	—	—	—	—
Deeg, 1992^48^	10-year LS	—	The Benton Visual Retention Test	—	—	—	—	—
Praetorius Bjork, 2016^50^	10-year LS	—	MRT, PR, TPM, IT (modified WAIS), DSF	—	—	BD, FLT	DSB	SDSST
Raji, 2005^49^	7-year LS	MMSE	—	—	—	—	—	—
Samper-Ternent, 2008^45^	10-year LS	MMSE	—	—	—	—	—	—
Taekema, 2012^53^	4-year LS	MMSE	12-PLT	—	—	—	Abbreviated Stroop Test	LDST
Veronese, 2016^57^	4-year LS	MMSE	—	—	—	—	—	—

CS, cross-sectional study; CSI-D, the community screening instrument of dementia; MMSE, mini-mental state examination; WMS, Wechsler memory scale; NAART, the North American adult reading test; TMT, trail making test; WCST, Wisconsin card sorting test; SCWT, Stroop color and word test; WAIS-R, Wechsler adult intelligence scale-revised; MoCA, Montreal cognitive assessment; DSF, digit span forward; DSB, digit span backward; PASAT, paced auditory serial addition test; LS, longitudinal study; 3MS, the modified mini-mental state examination; LM, logical memory; EBS, the east Boston story; WLM, word list memory; WLRc, word list recall; WLRcg, word list recognition; BNT, the Boston naming test; VF, verbal fluency; RT, reading test; JLO, judgement of line orientation; SPM, standard progressive matrices; DO, digit ordering; SDMT, symbol digit modalities test; NC, number comparison; SNST, Stroop neuropsychological screening test; CASI, Cognitive Abilities Screening Index Instrument; MRT, the memory-in-reality test; PR, prose recall; TPM, Thurstone’s picture memory; IT, information test; BD, block design; FLT, the figure logic task; SDSST, speeded digit-symbol substitution test; 12-PLT, 12-Picture Learning Test; LDST, Letter Digit Substitution Task.

### Cross-sectional studies

#### Handgrip strength and cognitive function

Six studies^32^^–^^37^ reported significant cross-sectional associations between cognitive performance and handgrip strength in community-dwelling older adults (Table 1), whereas one study^31^ did not find any association. The studies reported on 7,335 healthy community-dwelling older adults with ages ranging from 49 to 100 years. The proportion of males was about 44.6%, excluding the study that included only women.^36^ Years of education varied greatly among studies, ranging from elementary school not completed to 1 year and over of undergraduate studies. The studies used different kinds of handgrip strength devices and different approaches to analyze the association between handgrip strength and cognitive performance.

A study using the MMSE and Animal Naming tests^34^ divided the cognitive data into tertiles according to their scores in each test. The study found that handgrip strength was significantly different among the three tertiles of both cognitive tests; that is, the lowest tertile of the cognitive performance in both tests was associated with the lowest grip strength. Another study divided the handgrip strength data into tertiles and found a significant correlation between lower handgrip strength and lower MMSE scores.^37^ Handgrip strength was also correlated with successful aging (SA), defined as an MMSE score of ≥24, along with two other measurements.^33^ Other studies^31^^,^^32^^,^^35^^,^^36^ subdivided their data into cognitively normal and impaired using the following cut-off scores: one study^35^ used the Chinese version of the Community Screening Instrument of Dementia (CSI-D) with a cut-off of 28.4, and the other three studies^31^^,^^32^^,^^36^ used the MMSE with a cut-off of less than 24 to determine cognitive impairment.

The studies using the full scores of the cognitive tests^32^^,^^35^^,^^36^ found that handgrip strength performance was significantly lower in the cognitively impaired subjects, whereas the study using a subtest of the MMSE,^31^ the 3-Word Delayed Recall, to differentiate cognitively impaired subjects found no significant association between cognitive performance and handgrip strength.

#### Hand dexterity and cognitive function

The four cross-sectional studies in this category^28^^,^^38^^–^^40^ reported an association between hand dexterity and cognitive performance (Table 1). The studies reported on 370 healthy community-dwelling older adults, with ages varying from 23 to 93 years. The proportion of males ranged from 28.6% to 70%, and years of education ranged from 8 to 17 years.

To assess hand dexterity, Yozbatiran et al^38^ utilized the nine-hole peg test (9-HPT), a short-time measurement of upper extremity function.^41^ This study found a significant association between hand dexterity and the Paced Auditory Serial Addition Test (PASAT), a cognitive test used to assess attention, processing speed, and working memory.^42^^,^^43^ The Grooved Pegboard Test (GPT), a short neuropsychological test, was used by two studies^39^^,^^40^ to assess hand dexterity. In these studies, GPT was analyzed as the time in seconds to complete the test, and the data was divided into right and left hands, with one study^40^ including a composite score (combined scores of right and left hands). In another hand dexterity study,^28^ dexterity was examined with a modified version of the Purdue Pegboard Test (PPT). Hand dexterity was analyzed by assessing the time, angular displacement, and angular velocity of the right hand when reaching, grasping, transporting, and inserting pins and washers.

The data shown in the present review includes only that of healthy older adults; however, it is important to mention that two of these studies^38^^,^^40^ included neuro-muscular disease patients in their measurements and found a significant association between hand dexterity and cognitive performance. The healthy older adults were the control groups in both of these studies.

### Longitudinal studies

Most of the longitudinal studies examining the association between handgrip strength and cognitive performance indicated a significant association (Table 2).^44^^–^^53^ The studies reported on 16,531 community-dwelling older adults, with ages ranging from 46 to 100 years. The proportion of males was about 57.8%, excluding the study that included only women.^52^ Years of education greatly varied among studies, ranging from elementary school not completed to 1 year and over of undergraduate studies.

Although the association between these two variables has been demonstrated, it is unclear which variable at baseline influences the other in the long-term. Some of the studies^48^^–^^50^^,^^52^^,^^53^ indicated that having a lower cognitive performance at baseline influences the changes in handgrip strength in the long-term. More specifically, Deeg et al^48^ reported that scores in the Benton Visual Retention test,^54^ a short-term memory test,^55^ predicted changes in handgrip strength over 10 years in Japanese older men, whereas no significant association was seen in women. Another study^49^ indicated that subjects with low baseline MMSE scores, who tended to be older, less educated, and more depressed, had a significantly greater decline in handgrip strength over 7 years than their counterparts, even after covariate adjustment, including age, sex, education, and medical conditions. In addition, a study evaluating various cognitive domains at baseline^50^ reported a significant association with handgrip strength decline in older adults aged 80 years and over. A similar finding was suggested in a study evaluating the association of baseline cognitive domains,^53^ such as global cognition, memory, attention, and processing speed, with handgrip strength. Finally, a study using the modified MMSE (3MS)^52^ found that low 3MS scores at baseline significantly predicted decline in handgrip strength in older women over time; on the other hand, having a lower handgrip strength at baseline did not predict any longitudinal changes in cognitive scores.

In contrast, other studies^44^^–^^47^^,^^51^^,^^53^ suggested that subjects with the lowest handgrip strength at baseline had significantly more cognitive decline and risk of MCI in the long-term than those with higher handgrip strength. In a 25-year follow-up study,^51^ handgrip strength was significantly associated with the Cognitive Abilities Screening Index Instrument (CASI), a cognitive test that measures various cognitive domains and is considered more sensitive to variations in cognitive impairment than the MMSE.^56^ On the other hand, other studies^44^^–^^47^^,^^53^ used the MMSE test to measure cognitive performance, with two of them^46^^,^^53^ also including different kinds of cognitive tests. Although the studies suggested an association between baseline handgrip strength and changes in MMSE^44^^,^^45^^,^^47^ and other cognitive variables,^46^ Taekema et al^53^ only found an association with MMSE changes and not with other cognitive variables, such as memory, attention, and processing speed.

Contrary to the findings discussed above, Veronese et al^57^ did not find a significant association between baseline handgrip strength and the onset of cognitive impairment. It is important to note that, while unadjusted values indicate a significant association (*P* < 0.001), the association was no longer significant after covariate adjustment for age, gender, years of education, body mass index, smoking, activities of daily living (ADLs), instrumental ADLs, physical activity, geriatric depression scale, and medical conditions.^57^

## DISCUSSION

This review considered observational studies investigating the association between cognitive performance and hand motor function, with special consideration to those that evaluated handgrip strength and hand dexterity. The findings indicate a significant association between handgrip strength and cognitive performance or hand dexterity and cognitive performance; however, no studies evaluated the association among the three variables. Therefore, the present review has been outlined according to the findings for handgrip strength and hand dexterity separately.

Most of the longitudinal studies evaluated the association between either baseline handgrip strength and cognitive performance changes or vice-versa; however, only two studies^52^^,^^53^ analyzed both directions of the association. Because it is unclear which variable (handgrip strength or cognitive performance) influences the other in the long-term, it is necessary to consider the baseline effects of each variable on the changes of the other variable of interest. This also applies to the association between hand dexterity and cognitive performance, in that their association was examined only cross-sectionally.^28^^,^^38^^–^^40^ It would help to evaluate the influence of baseline hand dexterity and baseline cognitive function on subsequent changes in these domains.

Regarding cognitive performance, about 63.6% of all studies considered global cognition in their measurements, with the MMSE as the preferred test, whereas 36.4% considered other cognitive domains (Table 3). The studies that evaluated global cognition seemed to focus on the onset of cognitive impairment as a predictor of the association with handgrip strength. In this regard, a cross-sectional study using a subtest of the MMSE to define cognitive impairment^31^ reported no association between global cognition and handgrip strength, suggesting that cognitive evaluation should not be limited to global cognitive performance but should also include complex cognitive domains. By doing this, it would be possible to elucidate what cognitive domains relate to handgrip strength and hand dexterity. Furthermore, the cognitive test employed to detect cognitive impairment in community dwellers must be appropriately selected. Contrary to the studies included in this review, some studies suggest using the MMSE for screening dementia and the MoCA test for screening MCI,^58^ whereas the CASI test is claimed to be more sensitive than the MMSE for screening dementia in other studies.^56^

Concerning hand motor function, all of the studies included in this review (Table 1 and Table 2) measured handgrip strength as part of a physical function test battery to evaluate whole-body muscle strength or as a composite score to discriminate frailty. The studies acknowledge the association between handgrip strength and global cognitive performance, while the association with other cognitive domains needs to be observed further. On the other side, tests of hand dexterity, whose completion requires high cognitive demand as well as complex sensorimotor coordination, such as eye-finger coordination,^28^^,^^30^^,^^59^ were evaluated not only in community-dwelling older adults but also in neuromuscular disease patients to evaluate fine motor coordination skills.^38^^,^^40^ Attention seems to be another key factor associated with hand dexterity performance,^38^^,^^39^ which may be of interest for further evaluation in community-dwelling older adults. Therefore, it may be implied that hand dexterity test scores reflect complex functional aspects of motor and cognitive demands, such as attention, speed, and coordination. This assumption is in accordance with a previous study^59^ suggesting that hand function should consider not only the assessment of grip strength but also the upper motor coordination and sensorimotor processing domains.

Another point to consider is the covariate adjustment. In this regard, Veronese et al^57^ found no significant association between handgrip strength and cognitive impairment after adjusting for covariates. It is important to note that, unlike the other longitudinal studies included in this review, this study considered health behavior covariates, such as smoking, drinking, and exercise, besides demographic covariates and medical conditions. This assumption is supported by Atkinson et al^52^ who found no association between baseline handgrip strength and cognitive performance changes, after statistical adjustment for health behavior covariates. This is, however, not supported by cross-sectional studies in that these variables did not affect the significance of the relationship. Unlike cross-sectional studies, longitudinal studies are better at analyzing certain pattern of behaviors and may suggest other factors that may influence the association of interest. Therefore, we suggest caution when using covariates, especially in long-term studies, in that they may change the interpretation of the results.

### Strengths and limitations of the review

The strengths of this systematic review include the examination of the integrated assessment of hand motor function (handgrip strength and hand dexterity) and its relation to cognitive function in community-dwelling older adults. This review also provides an understanding of the current methodological approaches used for examining the association in question. However, the findings of the current review need to be interpreted with caution. First, we only examined studies that considered handgrip strength and hand dexterity as variables of hand function. Second, we have only searched articles written in English. Third, we did not perform a risk of bias assessment of the studies included in this review. Despite of these limitations, this review contributes to our understanding of the complexity of hand function as a sensorimotor and cognitive integrated mechanism and acknowledges hand motor function as a measurable indicator for the early detection of cognitive impairment in healthy older adults.

### Implications for future study

Unlike handgrip strength, only cross-sectional studies evaluated the association between hand dexterity and cognitive performance. We suggest analyzing this association in the long-term to elucidate the ways in which hand dysfunction may be related to cognitive impairment. Also, most of the cross-sectional studies evaluating hand dexterity included small sample sizes (less than 50); therefore, it is necessary to increase the sample size in community dwellers to make it more representative. We also recommend grouping the samples into narrower age groups (ie, 70–74, 75–79 years old), especially if they come from a small sample size, considering that cognitive performance may be more vulnerable to changes as we age.^60^ Finally, by analyzing the cross-sectional and longitudinal associations between hand dexterity, handgrip strength, and cognitive domains in older adults, there would be a better understanding of the mechanisms related to cognitive decline in late adulthood.

### Conclusions

Handgrip strength associates with cognitive performance cross-sectionally and longitudinally; however, it is unclear to speculate which variable—having weak handgrip strength or having low cognitive performance at baseline—affects the other in the long-term. Hand dexterity associates with cognitive performance cross-sectionally in community-dwelling older adults. Most of the studies utilized global cognition as an indicator of cognitive performance; therefore, long-term studies measuring different cognitive domains, as well as hand dexterity tests other than pegboard tests, such as line tracing and hand steadiness, are needed.
